# Detection of LV function abnormality using temporal patterns of normalized wall thickness

**DOI:** 10.1186/1532-429X-17-S1-P47

**Published:** 2015-02-03

**Authors:** Mai Wael, El-Sayed Ibrahim, Ahmed S Fahmy

**Affiliations:** 1 University of Michigan, Ann Arbor, MI, USA; 2 Nile University, Cairo, Egypt

## Background

Global measures of cardiac function may not reflect subtle wall motion abnormalities. In such cases, assessment of regional cardiac wall motion is required, which is usually evaluated visually with highly subjective results. Automatic analysis techniques can be classified into intensity-based and contour-based methods with the latter category avoiding the image quality-related limitations in the first one. In this study, we present a contour-based technique for detecting wall motion abnormality based on studying the temporal pattern of normalized wall thickness.

## Methods

**Figure 1:**
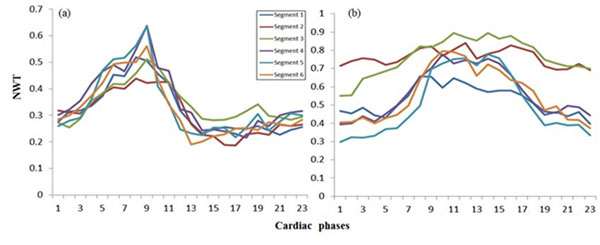
Normalized wall thickness (NWT) throughout the cardiac cycle for all six segments in a mid-ventricular short-axis slice from (a) normal (volunteer) and (b) patient with hypertrophy.

Image datasets from 14 volunteers and 13 patients (4 with myocardium infarction, 5 with pulmonary hypertension; and 4 with myocardium hypertrophy) were used to train and test the proposed classifier. Three short-axis cine images were acquired at basal-, mid-, and apical sites. All images were manually segmented to extract the epi- and endocardium. Each contour was re-sampled at equi-angular spaces to vectors of 60-points in the mid and basal slices and 40-points in the apical slices. Regional temporal changes in wall thickness were extracted based on standard AHA 17-segment model. The extracted thickness pattern was normalized relative to average epicardial radius. The wall thickness values of all points within each segment were averaged to one value (Fig. 1).

The *1-fold leave-one-out* method [1] was applied for training the classifier. Principal-component analysis was applied to find the directions of data variations and for dimensionality reduction. F1-score was used to select the proper number of principal-components. Naïve Bayes' classifier was applied to assign label to each segment (normal or abnormal). Slice abnormality was determined if slice contains two or more abnormal segments.

## Results

**Figure 2:**
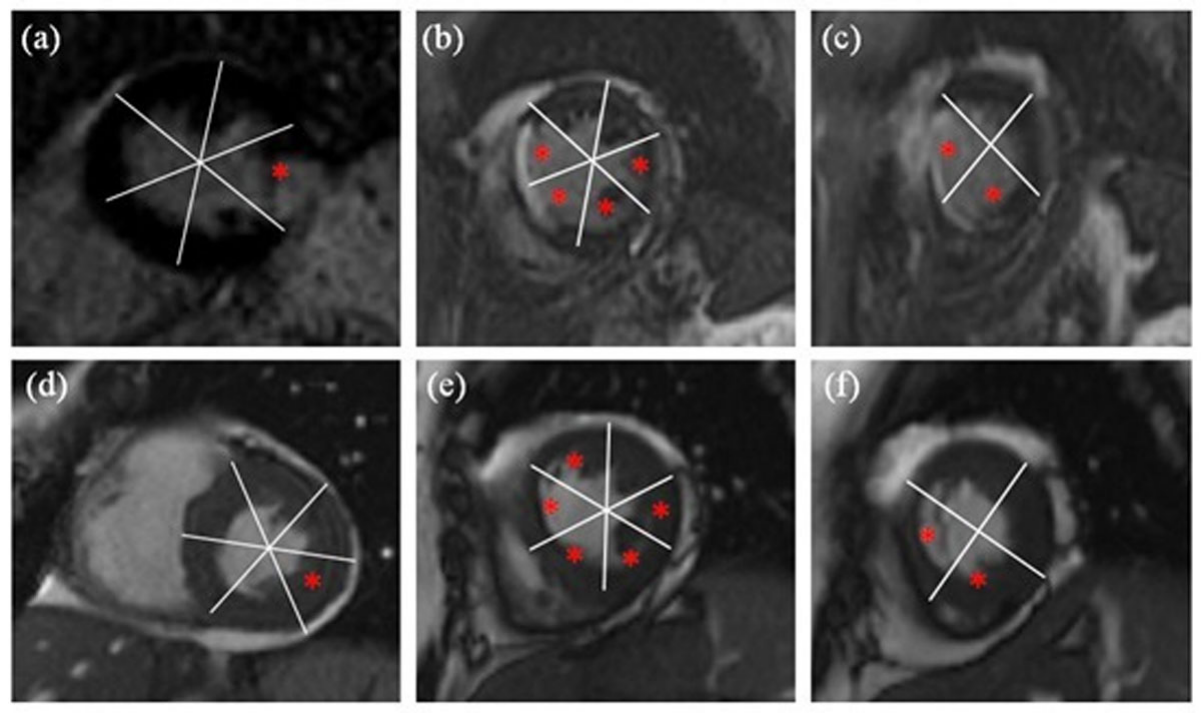
Comparison between infarcted regions using delayed-hyperenhancement MRI (a,b, and c) and regions with motion abnormality detected using the proposed method (d,e, and f).

The highest F-score (highest accuracy) occurred when one principal-component was used, which captures 89% of all data variations. 100% true-negative and 70% true-positive were achieved for basal- and mid-slices and 70% true-positive for apical-slices. Higher specificity relative to sensitivity was recorded, which reflects that the algorithm's tendency for true identification of normal cases over abnormal cases. The overall system accuracy was 88%, 88%, and 85% for basal, mid, and apical slices, respectively. Fig. 2 shows the resulting high agreement between the classifier results and ground-truth viability images.

## Conclusions

The proposed method provides automatic assessment of regional myocardial abnormality in a segmental basis for each slice; therefore, it could be a valuable tool for automatic and fast detection of early signs of cardiac dysfunction from conventional untagged cine images.

## Funding

N/A.
